# Sub-grouping and sub-functionalization of the RIFIN multi-copy protein family

**DOI:** 10.1186/1471-2164-9-19

**Published:** 2008-01-15

**Authors:** Nicolas Joannin, Saraswathi Abhiman, Erik L Sonnhammer, Mats Wahlgren

**Affiliations:** 1 Department of Microbiology, Tumor and Cell biology (MTC), Karolinska Institutet, SE-17177 Stockholm, Sweden and Swedish Institute for Infectious Diseases Control, SE-17182 Stockholm, Sweden; 2 Stockholm Bioinformatics Center, AlbaNova University Center, Stockholm University, SE-106 91 Stockholm, Sweden; 3 Computational Biology Branch, NCBI, NLM, NIH, Bethesda, MD 20894, USA

## Abstract

**Background:**

Parasitic protozoans possess many multicopy gene families which have central roles in parasite survival and virulence. The number and variability of members of these gene families often make it difficult to predict possible functions of the encoded proteins. The families of extra-cellular proteins that are exposed to a host immune response have been driven via immune selection to become antigenically variant, and thereby avoid immune recognition while maintaining protein function to establish a chronic infection.

**Results:**

We have combined phylogenetic and function shift analyses to study the evolution of the RIFIN proteins, which are antigenically variant and are encoded by the largest multicopy gene family in *Plasmodium falciparum*. We show that this family can be subdivided into two major groups that we named A- and B-RIFIN proteins. This suggested sub-grouping is supported by a recently published study that showed that, despite the presence of the *Plasmodium *export (PEXEL) motif in all RIFIN variants, proteins from each group have different cellular localizations during the intraerythrocytic life cycle of the parasite. In the present study we show that function shift analysis, a novel technique to predict functional divergence between sub-groups of a protein family, indicates that RIFINs have undergone neo- or sub-functionalization.

**Conclusion:**

These results question the general trend of clustering large antigenically variant protein groups into homogenous families. Assigning functions to protein families requires their subdivision into meaningful groups such as we have shown for the RIFIN protein family. Using phylogenetic and function shift analysis methods, we identify new directions for the investigation of this broad and complex group of proteins.

## Background

Antigenic variants are proteins expressed by pathogenic organisms, which are usually exposed to immune pressure from a vertebrate host. The genes that encode these proteins can be single copy within the genome as is the case for viruses and the variability therefore exists between gene copies of individuals. This implies that the proteins they encode retain the same function. However, other organisms maintain several to many copies within the genomes of each individual [1,2]. Conversely to viral genes, these multicopy genes are not only under immune pressure but can also follow distinct evolutionary paths to differentiate into novel functional units.

The genomes of *Plasmodium *species contain numerous large multigene families that have been amplified via functional or immune pressures [2-6]. One important feature of these organisms is that they do not express the whole protein repertoire simultaneously [7-10]. These polymorphic families are predominantly situated in the sub-telomeric ends of chromosomes [2-6], where gene rearrangements are frequent [11,12]. They encode for proteins that presumably fulfill several functions and immune pressure has driven them to antigenically vary at the surface of the infected erythrocyte [13]. Empirical studies have shown that the *Plasmodium falciparum *Erythrocyte Membrane protein 1 (PfEMP1) can mediate cytoadhesion by interacting with various host receptors, resulting for example in sequestration of the infected erythrocytes in the host tissue or rosette formation with uninfected red blood cells [13]. The repertoire of PfEMP1 proteins is therefore shaped both by functional pressures for binding and by diversifying pressures to evade immunity [14]. Yet, such an accumulation of experimental data is missing for protein families in most parasite species.

We have studied the RIFIN protein family, a group suggested to be under immune diversifying selection. Their genes, *repetitive interspersed family *(*rif)*, are the largest family in *P. falciparum *with 150 to 200 copies per haploid genome. They are small two-exon genes (≈1000 base pairs), with a conserved domain architecture [15,16]. Characteristically, RIFIN proteins are described as small polypeptides beginning with a putative signal sequence followed by a conserved domain, a variable region and a conserved C-terminal domain. Two transmembrane regions have been predicted on both sides of the variable region; with this stretch predicted to be exposed to immune pressure [9,15]. The proteins most closely related to RIFINs are of the Sub-Telomeric Variable Open Reading Frame (STEVOR) family [15], numbering 28 copies in the reference strain genome [2]. Although primary sequence similarity is limited [15], this relationship is emphasized by the existence of a *RIFIN_STEVOR *family (PF02009) in the PFAM database [17].

RIFIN proteins have been detected throughout the intra-human life cycle of the parasite [8,18-21]. Furthermore, RIFIN proteins are associated with a stable immune response over time and with rapid clearance of parasites from the circulation [22,23]. However, as for most protein families, little more is known and their function(s) remain(s) to be discovered. In this study, we propose a novel approach to understand complex protein families for which little data is available. We demonstrate the division of the RIFIN family into two groups, which we associate with published differential cellular localization. Finally, we correlate these differences with the prediction of a function shift between these sub-groups.

## Results

### Phylogenetic classification of the RIFIN family

**Figure 1:**
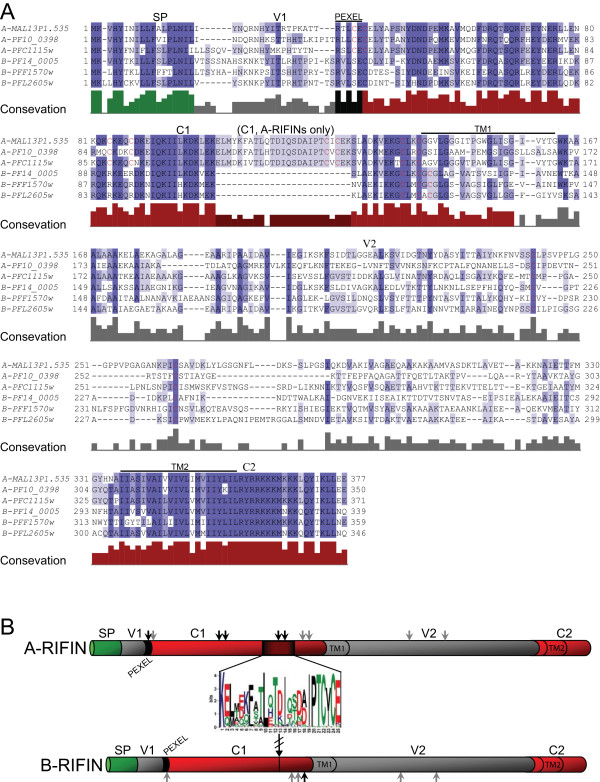
**RIFIN proteins overview**. (A) Alignment of a selection of A and B-type RIFINs. Conserved cysteines are highlighted in red; shading according to conservation. (B) Schematic RIFIN sub-group characteristics: Overall domain organization and classification into subtypes. The 25 amino acid stretch present only in the semi-conserved domain of A-type RIFINs is highlighted and depicted by a sequence logo. Grey arrows: common conserved cysteine residues; black arrows: sub-type specific cysteine residues; SP: signal peptide; PEXEL: *Plasmodium *export element; C1: semi-conserved domain, including the 25 AA insertion/deletion; C2: C-terminal conserved domain; TM1 and TM2: previously predicted transmembrane regions; V1: first variable domain; V2: second variable domain.

An alignment of 134 RIFIN protein sequences from the *P. falciparum *reference strain 3D7 (selection criteria detailed in Methods) was analyzed in order to detect divergences within the family. This revealed the existence of differences, prompting an initial division of RIFIN proteins into at least two major groups. The larger group, which we named the A-type RIFINs, represents ≈72% (97/134) of all analyzed proteins, while the second group, which we designated B-type RIFINs, makes up ≈28% (37/134). Although both groups have a common architectural structure [15,16], they differ by several features, as depicted in the alignment of representative A- and B-RIFIN sequences (Fig. 1A) and schematically (Fig. 1B). First, the A-type proteins are generally larger than the B-type variants (on average 350 and 330 amino acids, respectively). This difference is largely due to a 25 amino acid stretch present only in the conserved (C1) region of A-type RIFINs, as previously described [2]. It is located approximately 66 amino acids downstream of the *Plasmodium *export element (PEXEL motif) [24] and contains some highly conserved residues (Fig. 1). A second distinctive feature concerns the number of conserved cysteine residues (Fig. 1B arrows). A-type RIFINs are characterized by a total of 10 highly conserved cysteine residues, compared to 6 in B-type variants, 5 of which are common to both sub-types (Fig. 1B grey arrows). Notably, two of the conserved cysteines typical for A-type RIFINs are found in the 25 amino acid stretch.

**Figure 2:**
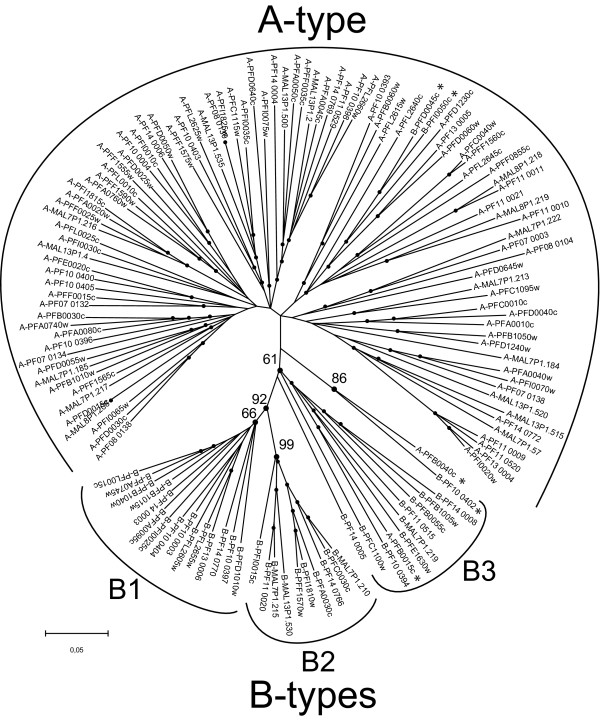
**Phylogenetic tree of *rif *cDNA**. The tree shows the segregation of A- and B-*rif *genes (gaps considered as complete deletions). The B-*rif *group is further subdivided into B1, B2 and B3 clusters. Stars indicate sequences that group atypically. Bootstrap support, after 1000 replicates, is only shown for the branches separating the different groups, dots at nodes indicate bootstrap values above or equal to 60%.

**Figure 3:**
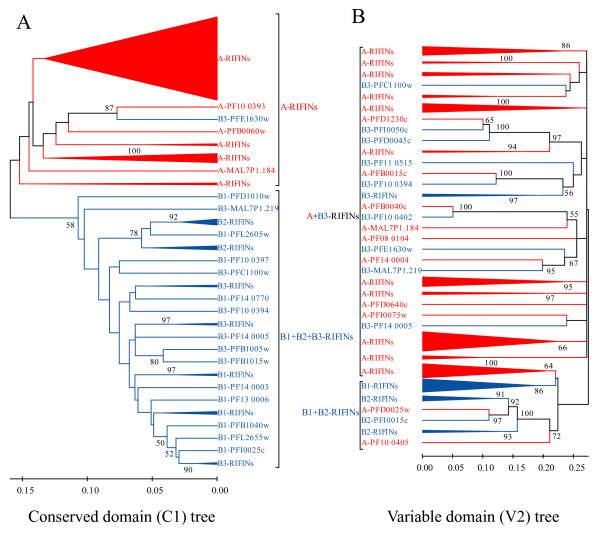
**Non-congruence of phylogenetic trees of RIFIN conserved (C1) versus variable (V2) domains**. (A) Neighbor Joining tree of the C1 domain (gaps considered as pairwise deletions) showing the segregation of A- from B-RIFIN sequences. (B) The same tree construction method applied to the V2 domain showing that B3-RIFIN sequences do not cluster with B1- and B2-sequences. Bootstrap support, after 1000 replicates, is shown for values above 50%.

In order to substantiate this preliminary sub-grouping, we clustered *rif *sequences according to their similarities by constructing Neighbor Joining distance trees. The trees resulting from protein-derived cDNA alignments sorted the sequences into two major groups that were largely concordant with the above sub-grouping (Fig. 2). However, five sequences deviate from their predicted group (Fig. 2, stars): PFD0045c and PFI0050w, which are B-RIFINs, cluster with A-RIFINs; PFB0015c is an A-type which groups with B-RIFINs; and PFB0040c and PF10_0402 cluster together and separately from A- or B-RIFIN proteins. We find it noteworthy that the B-RIFIN group could be further subdivided into three subsets, namely B1, B2 and B3, whereas the A-RIFINs did not form any obvious clusters (Fig. 2). While B1 and B2 sub-clades formed a monophyletic group with a bootstrap value of 92%, the separation of the B clade from the A clade had a weaker statistical support at 61%. This unexpectedly low bootstrap value together with the observation of relatively long branches in the B3 sub-group versus the shorter ones in the B1 and B2 sub-groups prompted us to examine the sequences more closely. Accordingly, we carried out independent phylogenetic analyses of the conserved C1 and the variable V2 domains (as shown in Fig. 1B). These trees show that the B3 sequences have an incongruent history (Fig. 3), which reveals probable recombination/gene conversion events. Specifically, the V2 domains of the B3 subset segregated with the A-RIFINs rather than with B-RIFINs, while the C1 domains of the same variants were of B-type (with the exception of PFE1630w). B3 sequences thus constitute hybrid variants composed of C1 domains of the B subtype and V2 domains of the A subtype. Overall, we observed long branches for sequences encoding A- and B3-RIFIN proteins, not seen for B1 and B2 sequences, clearly a direct consequence of the higher variability within the V2 region of these sequences.

In addition to the analysis of the 3D7 strain, we have aligned the 3D7 sequences with 59 of the DD2 and 65 of the HB3 strain sequences (selection criteria detailed in Methods). The tree resulting from the protein alignment confirmed the results obtained with the reference genome analyses. The sequences sorted into the same two major clades with no strain specific grouping (see Additional file 1). The B-RIFIN clade is split into three groups; however the B1 and B2 clades contain few sequences from the DD2 and HB3 genomes.

It is noteworthy that the two B-RIFIN sequences, which cluster with A-RIFINs (PFD0045c and PFI0050w), have homologous sequences in both DD2 and HB3 genomes (see Additional file 1, stars).

Based on the knowledge that non-coding regions may contain motifs of significance in gene regulation and expression, we also analyzed 500 base pairs of non-coding upstream and downstream untranslated regions (UTRs) from the 3D7 *rif *genes. The phylogenetic analyses of these regions segregated the sequences into the same major A- and B- groups as the coding regions, which we have termed *A-rif *and *B-rif *UTRs (see Additional file 2). For both 5' and 3' UTR analyses, *B-rif *UTRs could be further divided into two groups, one of which included B1 and B2 variant UTRs, the other mostly B3 variant UTRs. As in the above analysis, some sequences did not segregate into their expected sub-group, for example a few B3 sequences were found in the B1/B2 subdivision and vice versa. Additionally, some *A-rif *UTRs clustered with *B-rif *UTRs and in this case, mostly with the B3 sub-group. In contrast to the coding sequences, the *A-rif *UTRs appear to cluster into sub-groups. Despite overall similarities in observations between both 5' and 3' UTR analyses, there was only partial congruence between these UTR clusters, in particular as far as *A-rif *UTRs are concerned.

A previous study has identified two transcriptional repression sites (TATGCAATGATT and CGCACAACAC) [25] upstream of 8 *rif *genes in a head to head orientation with UpsA *var *genes. An exhaustive search on all 14 chromosomes of the 3D7 strain shows that these two motifs are found in 20 and 19 copies, respectively. However, only 15 and 11 copies are upstream (either independently or in combination) of a total of 16 *rif *genes (see Additional file 2, indicated by #); the other copies are found up- or downstream, or sometimes in the coding region of other genes. Concordantly to this analysis, 13 of the 5' UTRs of these genes cluster together in our phylogenetic tree.

An analysis of chromosomal location reveals that only 6 of the 134 sequences (4.5%) used in this study are centrally located genes (data not shown). The other similarly positioned *rif *genes are annotated as pseudogenes or are truncated and none of these are grouped according to protein or UTR sequences (data not shown). The transcription of ≈70% of *A-rif *and all *B-rif *genes is telomere oriented. The *A-rif *genes with a centromeric transcription orientation (≈30%) do not cluster on the protein tree (data not shown), however they are mostly distributed within three sub-clades of the *A-rif *5' UTR tree (see Additional file 2, crosses).

### Function shift analysis of A- and B-RIFIN proteins

We sought for indications of functional differences between A- and B-RIFIN sub-groups by analyzing them for function shifts according to previously described methods [26]. Function shift analysis calculates the number of rate and conservation shifting sites (RSS and CSS, respectively) that exist between two given protein groups. RSS is measured by U-values, which indicate the likelihood that the mutation rate changes for each alignment position between the subfamilies under consideration. A site is considered rate-shifting (at 5% significance level) if its U-value is above a cut-off value of 4.0 [27]. CSS is measured by the *Z*-score, a normalized method to examine the similarity between two distributions of amino acids. Smaller *Z*-score values are associated with similar amino acid distributions in both subfamilies, while larger *Z*-score values are associated with very different distributions. The total numbers of positions are counted for both RSS and CSS calculations.

The results are compared to enzymatic protein families that have undergone a change in function, which belong to several functional categories including immunity related functions. The function shift model was benchmarked using organisms from all three kingdoms of life, namely Archea, Bacteria and Eukaryotes. This results in the estimation of the likelihood of sub-functionalization between the two groups. The function shift analysis of sub-group A against sub-group B (using standard cut-offs of 4 for RSS and 0.5 for CSS) resulted in the prediction of 81 rate shifting sites (RSS) (22% of all positions) and 60 conservation shifting sites (CSS) (17%) between them (see Additional file 3, rifins.html, for the full alignment). We computed the probability of the prediction as 83% based on RSS alone and 52% based on CSS alone. Considering comparable knowledge empirically gathered on the classification of shifts in function of known protein families, which combine the two measures [26], A- and B- sub-groups are predicted to have functionally diverged from each other.

**Figure 4:**
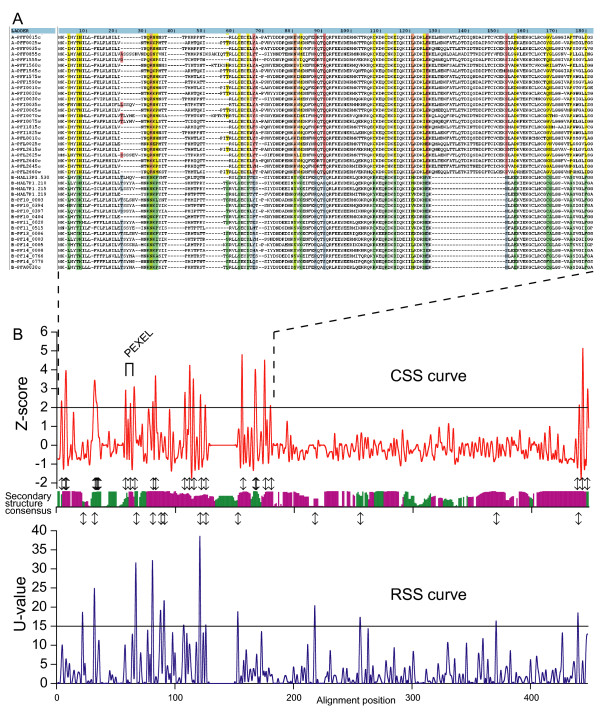
**Function shift analysis of A- and B- RIFIN proteins**. (A) Sample sequences from the high stringency global alignment available as Additional file 4. Columns with Orange-Blue represent RSS; columns with yellow-green represent CSS; columns with Salmon-green represent both RSS and CSS. (B) Plots of Z-scores and U-values, for CSS (red curve) and RSS (blue curve) respectively, according to alignment position. The predicted consensus secondary structure is plotted with pink and green bars representing helices and loops, respectively. The heights of the bars indicate conserved predictions. Arrows correlate the highest scoring shifted sites with secondary structure predictions.

Listed in Table 1 and 2 are the top positions sorted according to their U-values for RSS (stringent cut-off of 15) and Z-scores for CSS (stringent cut-off of 2), respectively. Both RSS and CSS are mostly found in the conserved regions of RIFIN proteins (see Additional file 4, rifins_high.html, for the full alignment with stringent cut-offs). In Figure 4A we show these shifts in a portion of the N-terminus of a random selection of A- and B-RIFIN sequences. Figure 4B correlates CSS and RSS plots, along the alignment, with the predicted conservation of secondary structure of RIFIN proteins. The high stringency cut-offs used in this figure highlight the most significantly shifted sites (Fig. 4B arrows). Notably, most of these shifts involve a change in the biochemical properties of the amino acid. We will specifically emphasize the shifts in positions Q31K, R32N, N33K and H34P, in a predicted loop region about 15 AA upstream of the PEXEL motif; positions C62S and Y67X approximately at the PEXEL motif; and positions C62S, C108R, C112R, and G167C which all involve cysteine residues, commonly engaged in disulfide bonds.

**Table 1:** Most significant Rate Shifting Sites

Position in the Alignment	Residues in A-RIFINs	Residues in B-RIFINs	U-value	Residue Conserved in family
121	L	HQVEKL	38.5120	A
81	EARASKD	S	32.1770	B
67	Y	EQPHYNR	31.5882	A
32	RKQWSNM	N	24.8960	B
91	QSTA	S	21.2050	B
218	A	TAQVIKL	20.3432	A
153	S	NSLK	18.7734	A
88	KRDLQVH	R	18.6986	B
22	X	T	18.6422	B
440	L	X	18.4488	A
256	X	S	16.4634	B
371	X	A	16.3382	B
126	E	DEVQ	15.3118	A

Top RSS positions sorted according to their U-values for RSS (stringent cutoff of 15).

**Table 2:** Most significant Conservation Shifting Sites

Position in the Alignment	Conserved Residue in A-RIFINs	Conserved residue in B-RIFINs	Z-Score
443	I	T	5.132
156	D	E	4.794
175	P	G	4.509
112	C	R	4.231
167	G	C	3.852
83	M	K	3.628
8	N	K	3.561
115	E	N	3.509
32	R	N	3.432
65	E	D	2.992
447	E	N	2.932
58	T	S	2.913
108	C	R	2.783
7	I	S	2.691
121	L	H	2.671
31	Q	K	2.442
33	N	K	2.423
4	V	L	2.347
81	E	S	2.287
62	C	S	2.211
168	I	G	2.144
440	L	A	2.127
125	L	M	2.122
180	I	F	2.093
34	H	P	2.061

Top CSS positions sorted according to their Z-scores (stringent cutoff of 2).

Limitations of function shift analyses lie in regions for which one group has amino-acid stretches that the other group lacks. In this case, RSS and CSS calculations give a null value; however this does not equate to an absence of impact on functional divergence of the two groups. One particular way of viewing such a site is to acknowledge it as a shifted site from a conserved motif to an absence of residues. The 25 AA stretch present in A-RIFIN sequences and absent from B-RIFINs can be viewed in this way, specifically due to the conservation of many of its residues as seen in Fig. 1B. Additionally, most of this motif is predicted to be a loop region, which could be involved in a functional site.

## Discussion

Protein families with known functions have successfully been sorted into functionally different sub-groups using phylogenetic techniques [28,29]. However, which approach should be used with proteins of unknown function? We have combined phylogenetic and function shift analyses to study the *Plasmodium falciparum *RIFIN protein family. Our results demonstrated that these proteins could be subdivided into two major groups that we named A- and B-RIFIN proteins. We correlate these groups with different localization studies [19,21,30] based on proteins from each of these groups. Moreover, our function shift analysis points to the probability that these two groups of proteins have undergone neo- or sub-functionalization.

The 3D7 *rif *cDNA tree we constructed by the Neighbor Joining method distinguished A- and B-type RIFIN variants, the latter being subdivided into three groups (B1, B2 and B3). The additional analysis of combined *rif *sequences from three different strains (3D7, DD2 and HB3) confirms this grouping (see Additional file 1). However, most DD2 and HB3 sequences clustered in the A and B3 groups, with only four sequences in the B1/B2 group. Our strict inclusion criteria have resulted in the removal of over 45% of the DD2 and HB3 RIFINs, mainly truncated sequences. We do not know whether these are simply pseudogenes within these genomes or if they appear as truncated due to the difficulties in sequencing and assembling subtelomeric regions of *P. falciparum *parasites. Considering this latter case, we prefer not to draw genome wide conclusions from possibly incomplete genomes.

Upon further investigation of the 3D7 RIFINs, B3-sequences showed to be hybrid variants that have B1/B2 features in their C1 domains but A-type features in their V2 domains. Vice versa, two A-variant hybrids carrying A-specific C1 domains and B1/B2-specific V2 domains were also found (Fig. 3). Recombination events and gene conversion are likely to serve as explanations for the formation of such hybrid sequences. The former are essential for the generation of antigenic diversity [11] and previously proposed to be responsible for the diversity of the *var *gene family [31]. These authors argue for recombination events restricted between genes grouped according to their chromosomal location and transcription orientation. In contrast to the *var *genes, there is no evidence for such specific recombination within the *A- *and *B-rif *gene groups: ≈70% of the *A-rif *and all *B-rif *genes have the same telomere-directed transcription orientation; the remaining ≈30% of *A-rif *genes do not cluster in our gene tree. Also, over 95% of all *rif *genes analyzed here are subtelomeric. Theoretically, recombination can thus occur between A- and B- types of the same orientation. DePristo *et al*. showed that low-complexity regions are preferred sites for recombination events to occur in *var *genes [32]. Since low-complexity regions are commonly found within RIFIN sequences at the boundaries of the variable region, it is tempting to suggest these sites to have a role in the generation of such hybrid sequences. Gene conversion has been observed in *P. falciparum *[11,33,34] and is the other possible explanation for these sequences. However, gene conversion has a homogenizing effect that is not detected between B3-*rif *V2 regions and the sequences showing highest identity to them (66,6% average sequence identity). This might be an indication in favor of recombination events or, simply, that gene conversion is not as frequent as suggested for *falcipain *genes [34].

Whichever mechanism, both recombination and gene conversion events are known to interfere with phylogenetic reconstruction [35]. Another factor that influences the resolution of phylogenetic analysis is long branch attraction [36,37]. We have seen that A- and B3-RIFIN sequences have long branches (Fig. 2), which could also interfere in our phylogeny. To further confirm our proposed sub-grouping, we constructed phylogenetic trees of the UTRs of *rif *genes. Previous analysis of gene families has shown that long-term survival of paralogous genes allows for changes in the regulatory regions of those genes [38]. Our analysis of *rif *gene UTRs demonstrated a significant segregation of these non-coding regions into similar *A- *and *B-rif *UTR groups (see Additional file 2). Taking all these facts into consideration, we conclude that despite a seemingly low bootstrap value of 61%, RIFIN proteins can be divided into A- and B-RIFIN proteins.

One question arises at this point: could there be an alternative grouping of *rif*/RIFIN sequences? *var*, the other major family in *P. falciparum *has been classified according to 5' UTR and genomic position [2,39,40]. Their classification into 3 major sub-groups (A ≈17%, B ≈42% and C ≈40%) mainly relies on the following features: (i) 5' UTR grouping (UPSA, B and C); (ii) gene position (A and B telomeric, C central); and (iii) transciption orientation (A and C towards the telomere, B towards the centromere) [39]. However, PfEMP1 proteins are more complicated than RIFINs by the fact they are modular. Recognizable signatures allow for the identification of each module but intra-module similarity is limited [2]. The overall function of these proteins is accepted as adhesion to host receptors and is highly module dependent (reviewed in [13]).

A parallel analysis of *rif *genes shows that, on one hand, very few are not sub-telomeric and no obvious pattern regroups these sequences. In the absence of more conclusive evidence, we do not think this is a good criterion for sub-grouping *rif *genes. On the other hand, *rif *UTR sequences can be grouped into sub-clusters. Also, the 5' UTRs of *A-rif *genes transcribed towards the centromere are non-randomly distributed (see Additional file 2, crosses). These observations confirm previous reports of differential regulation of *A-rif *expression within the same parasite strain [21]. However the clustering of these *A-rif *UTR sequences is not congruent with the clustering of the protein-derived cDNA sequences. A recent study of *yir *genes, the largest *P. yoelii yoelii *multigene family, shows that some *yir *genes undergo alternative splicing events [41], which implies regulatory signals in addition to those controlling gene activation and silencing. Therefore, although it is tempting to further the sub-grouping of *A-rif *genes, we believe additional experimental evidence of differential transcription is required to ascertain these sub-divisions.

A recent study has shown that the intracellular distribution of RIFIN molecules in the infected erythrocyte is more diverse than previously envisaged [21]. In order to address the issue of cross reactivity of the antisera used in this study, Petter *et al*. [21] tested recognition of the anti-RIF29 and anti-PFI0050c antisera against other recombinant proteins of each group. Also, their western blot analyses show that neither A-RIFIN antisera are cross-reactive. A-type RIFINs, detected by an antiserum directed against PFB1035w [8] as well as an antiserum directed against RIF29 [23] (both A-type RIFINs), are transported to Mauer's clefts and towards the surface of the infected cell [19,21], while B-type RIFINs, detected by an antiserum directed against PFB1040w [8] and an antiserum directed against PFI0050c [30] (both B-type RIFINs), are expressed inside the parasite [21], which is consistent with this group's previous report [30]. Additionally, both A- and B-RIFIN proteins were detected in merozoites, here again with different sub-cellular distributions [21]. The localization of B-RIFINs is concordant with the lower variability they exhibit in their V2 region, at least for the B1- and B2- RIFIN proteins (shorter branch lengths in Fig. 2). This would be expected of sequences not exposed to the immune system for long periods of time, as they would be at the infected erythrocyte surface.

Although all RIFIN variants bear a motif for directing proteins onto the secretory route, out of the parasite and into the cytoplasm of the host cell, referred to as the *Plasmodium *Export Element (PEXEL) or Vacuolar Transport Signal [24,42], additional factors not yet characterized might enhance or interfere with protein export. Bioinformatics analyses of biochemical properties of the PEXEL motif and surrounding amino acids suggest possible modulations of the role of this motif (J. Hiss, J. Przyborski, F. Schwarte, K. Lingelbach and G. Schneider, personal communication). Alternatively, presence or absence of conserved motifs distributed elsewhere in the protein, such as the 25 AA stretch present in A-RIFINs, and/or different native 3D conformations of A- and B-RIFIN variants due to the highly conserved subtype specific cysteine residues (possibly involved in disulfide bonding), could impose restrictions on the export signal carried by the PEXEL motif. A previous study of synthetic constructs of the gene PFI0050c (a B-RIFIN) fused to a green fluorescent protein shows that this protein is retained in the parasite when its full length is expressed [30]. However truncated versions, notably when lacking the C-terminal conserved region, are exported to the Maurer's Clefts. It is not clear whether this difference of localization is due to missing motifs in the C-terminus or to changes in 3D conformation due to the truncation of the C-terminus, including a transmembrane domain, of the protein. Whichever their respective transport mechanism, A- and B-RIFIN proteins have a distinct pattern of distribution during the intraerythrocytic life cycle of the parasite, which in correlation with the divergence of their regulatory regions [38] is suggestive of functional differences.

To test this hypothesis, we carried out a function shift analysis [26] of our sub-groups. The evolution of protein families and the consequential evolution of their function are accompanied by the accumulation of mutations at individual sites throughout the protein sequence [43]. These sites may incur different types of selective pressures. A specific site may become important for the maintenance of the function, and therefore a specific amino acid is fixed in that position. In contrast, a fixed site may lose its importance, and become prone to mutation (typical RSS sites). Alternatively, a switch of functional specificity of a site may result in the switch from one amino acid to another accompanied by strict conservation (no further mutations allowed) in both sub-groups (typical CSS site). Finally, the remaining mutations are thought to be randomly accumulated at selectively neutral sites. However, recent studies have shown that mutations in non-essential residues can greatly influence protein stability and aggregation [44]. These types of mutations may build up a compensation mechanism for mutations in key functional sites. Our function shift analysis shows, between A- and B-RIFIN proteins, which sites are under strict or varying selective pressure (see Additional file 3, rifins.html). Although the function shift analysis does not take into consideration sites for which one of the groups has a full gap (as the 25 AA insertion/deletion in the C1 domain), the accumulation of these shifted sites throughout the RIFIN sequences resulted in the prediction of a function shift between A- and B-type RIFIN proteins. A more stringent analysis of these shifted sites (see Additional file 4, rigins_high.html) identified specific residues about 15 AA ahead of and within the PEXEL motif with significant physical and chemical property changes. This analysis confirms the observations made by Hiss *et al*. (J. Hiss, J. Przyborski, F. Schwarte, K. Lingelbach and G. Schneider, personal communication). Also, the changes in cysteine conservation between the two groups are potentially involved in the variation of their three dimensional structures. These changes are likely to modulate the trafficking properties of RIFIN proteins. These predicted RSS and CSS sites can be tested, in future studies, by experimental techniques like site directed mutagenesis for their ability to bring about function changes.

Although *rif *genes have been initially discovered and subsequently studied in the blood stage of the parasite's life cycle [8,9,19,21,30,45], recent large scale transcriptional and proteomic analyses show that *rif *gene transcripts and RIFIN proteins are most abundant in sporozoites (25 and 20 respectively) as well as being present in gametocytes and merozoites [18,21,46-49]. Recent work in other *Plasmodia *species has also put forward modulations of expression and function of multi-copy protein families such as VIR of *P. vivax *and both YIR and PY235 of *P. yoelii yoelii *[41,50,51]. In particular, the expression of these proteins in different stages of the parasite life cycle advocates for a greater subdivision of these families and their specific functions.

## Conclusion

So far, the RIFIN protein family has been considered to be one large family with an unknown function but our results argue for a cautious approach when studying such variable protein families. The RIFIN proteins have been long neglected, possibly in part because of the complexity involved in studying such a large group of proteins. Antigenic variation is mostly a secondary function, as seen with the PfEMP1 proteins, which main function is in cytoadhesion. While physiological functions of RIFIN proteins remain obscure, it is expected that future focus on RIFIN sub-families, the 25 AA insertion/deletion and the predicted conservation-shifted sites between these sub-groups will help to simplify the quest for understanding their biological roles in the parasite. Finally, the lower variability of B-RIFIN molecules and their expression throughout the cycle of the parasite (multi-stage) suggest these proteins as candidate vaccine targets. Further analysis of this family in wild isolates may confirm this hypothesis.

## Methods

### Phylogenetic analysis and sequence representation

3D7 RIFIN sequences were retrieved from PlasmoDB v4.4 [52]; DD2 and HB3 sequence and annotation information was downloaded from the Broad Institute of Harvard and MIT [53]. Protein multiple sequence alignments were generated using the Kalign software [54] and manual refinement was carried out with the help of the BioEdit software [55]. We chose as inclusion criterion for RIFIN sequences that they correspond to the described *rif *and RIFIN structures: two exon gene and protein composed of a signal peptide followed by a conserved domain, a variable region and ending with a typical positively charged C-terminus. Out of the 159 RIFIN sequences from the 3D7 reference strain, 25 were either truncated sequences or lacked obvious similarity with the majority of RIFIN sequences and were thus eliminated from our analysis. Similarly, only 59 (of the 156 with a RIFIN_STEVOR PFAM annotation, 25 of which are STEVORs) and 65 (of the 131, 26 of which are STEVORs) sequences of DD2 and HB3, respectively, were retained for analysis.

Independent alignments and phylogenetic analyses were carried out, on one hand, for the 3D7 strain (134 sequences) and, on the other hand, for the combined 3D7, DD2 and HB3 strains (258 sequences).

Five hundred base pairs of upstream and downstream untranslated regions (UTR) as well as the cDNA sequences of the 3D7 rif genes were retrieved from GeneDB [56]. The UTRs were aligned in the same manner as the protein sequences.

Protein sequences are easier to accurately align than cDNA, however the degeneracy of the genetic code makes cDNA more informative than the corresponding protein translation. We used cDNA alignments derived from our protein multiple sequence alignments in order to increase the precision of the phylogenetic analysis. The cDNA alignments were constructed by replacing the amino acids in the protein alignments with the corresponding *P. falciparum *gene specific codons using the PAL2NAL software [57]. All the alignments are available upon request to the authors.

The C1 domain starts at the PEXEL motif and ends 30 AA after the insertion/deletion. The V2 domain starts 31 AA after the insertion deletion and ends 57 AA before the N-terminus of the protein alignment.

The alignments were used to construct distance trees using the Neighbor Joining method with the MEGA3.1 software [58]. We used a p-distance model with gaps/missing data treated as pairwise deletion for the proteins and UTRs and complete deletion for cDNA alignments. No trees were cut down throughout the experiments. In order to estimate robustness, bootstrap proportions were computed after 1000 replications.

Protein motifs were generated using Protein Sequence Logos and Relative Entropy server [59,60].

Secondary structure predictions were computed using PSIPRED [61,62]. The predicted secondary structures were aligned according to the protein alignment and a consensus prediction was generated using the Jalview software [63].

### Function shift analysis

The function shift analysis was carried out on each subfamily pair, of the 3D7 genome sequences (after exclusion of two A-RIFIN and four B-RIFIN sequences which are hybrid A/B sequences; see Discussion for further details), using a previously described method [26]. In this method, two types of sites, namely rate shifting sites [27] and conservation shifting sites [26] are detected and a combined measure is calculated to assess the level of function shift between the sub-groups under consideration. In order for the algorithms to calculate shifting sites, the sequences need to segregate into their predicted groups. Six sequences (two A-RIFIN and four B-RIFN proteins) clustered in the opposite sub-group creating systematic errors in the algorithm. These sequences are all hybrids and were excluded from the function shift analysis.

## Abbreviations

Amino Acid (AA), Conservation Shifting Site (CSS), *Plasmodium *EXport ELement (PEXEL), *Plasmodium falciparum *Erythrocyte Membrane Protein 1 (PfEMP1), Rate Shifting Site (RSS), repetitive interspersed family (*rif*), UnTranslated Region (UTR)

## Authors' contributions

NJ conceived of and designed the study; he performed the phylogenetic analysis and analyzed all data; he drafted and revised the manuscript. SA contributed in the design of the study, carried out the function shift analysis and contributed to data analysis and reviewing of the manuscript. ELS contributed to the data interpretation and to the reviewing of the manuscript. MW contributed to the conception of the study and reviewing of the manuscript. All authors read and approved the final manuscript.

## Supplementary Material

Additional file 1 Phylogenetic tree of 3D7, DD2 and HB3 *rif *genes. The Neighbor Joining tree shows the segregation of A- and B-*rif *sequences (gaps considered as pairwise deletions). Stars show atypically grouped B-RIFIN sequences from all three strains. Colours: 3D7 sequences in red; DD2 sequences in blue; HB3 sequences in green. Bootstrap support, after 500 replicates, is only shown for major branches, dots at nodes indicate bootstrap values above or equal to 50%. Click here for file

Additional file 2 Phylogenetic tree of 5' and 3' UTR sequences. The trees show the segregation of A- and B-*rif *UTRs (gaps considered as pairwise deletions). Bootstrap support, after 1000 replicates, is only shown for major branches, dots at nodes indicate bootstrap values above or equal to 60%. Click here for file

Additional file 3 Protein sequence alignment. Alignment of RIFIN proteins with function shifted sites at standard cut-off stringency. Columns with Orange-Blue represent RSS; columns with yellow-green represent CSS; columns with Salmon-green represent both RSS and CSS. Click here for file

Additional file 4 Protein sequence alignment. Alignment of RIFIN proteins with function shifted sites at high cut-off stringency. Columns with Orange-Blue represent RSS; columns with yellow-green represent CSS; columns with Salmon-green represent both RSS and CSS. Click here for file

## References

[B1] Stringer JR, Keely SP (2001). Genetics of surface antigen expression in Pneumocystis carinii. Infect Immun.

[B2] Gardner MJ, Hall N, Fung E, White O, Berriman M, Hyman RW, Carlton JM, Pain A, Nelson KE, Bowman S, Paulsen IT, James K, Eisen JA, Rutherford K, Salzberg SL, Craig A, Kyes S, Chan MS, Nene V, Shallom SJ, Suh B, Peterson J, Angiuoli S, Pertea M, Allen J, Selengut J, Haft D, Mather MW, Vaidya AB, Martin DM, Fairlamb AH, Fraunholz MJ, Roos DS, Ralph SA, McFadden GI, Cummings LM, Subramanian GM, Mungall C, Venter JC, Carucci DJ, Hoffman SL, Newbold C, Davis RW, Fraser CM, Barrell B (2002). Genome sequence of the human malaria parasite Plasmodium falciparum. Nature.

[B3] del Portillo HA, Fernandez-Becerra C, Bowman S, Oliver K, Preuss M, Sanchez CP, Schneider NK, Villalobos JM, Rajandream MA, Harris D, Pereira da Silva LH, Barrell B, Lanzer M (2001). A superfamily of variant genes encoded in the subtelomeric region of Plasmodium vivax. Nature.

[B4] Fischer K, Chavchich M, Huestis R, Wilson DW, Kemp DJ, Saul A (2003). Ten families of variant genes encoded in subtelomeric regions of multiple chromosomes of Plasmodium chabaudi, a malaria species that undergoes antigenic variation in the laboratory mouse. Mol Microbiol.

[B5] Janssen CS, Phillips RS, Turner CM, Barrett MP (2004). Plasmodium interspersed repeats: the major multigene superfamily of malaria parasites. Nucleic Acids Res.

[B6] Sam-Yellowe TY, Florens L, Johnson JR, Wang T, Drazba JA, Le Roch KG, Zhou Y, Batalov S, Carucci DJ, Winzeler EA, Yates JR (2004). A Plasmodium gene family encoding Maurer's cleft membrane proteins: structural properties and expression profiling. Genome Res.

[B7] Chen Q, Fernandez V, Sundstrom A, Schlichtherle M, Datta S, Hagblom P, Wahlgren M (1998). Developmental selection of var gene expression in Plasmodium falciparum. Nature.

[B8] Fernandez V, Hommel M, Chen Q, Hagblom P, Wahlgren M (1999). Small, clonally variant antigens expressed on the surface of the Plasmodium falciparum-infected erythrocyte are encoded by the rif gene family and are the target of human immune responses. J Exp Med.

[B9] Kyes SA, Rowe JA, Kriek N, Newbold CI (1999). Rifins: a second family of clonally variant proteins expressed on the surface of red cells infected with Plasmodium falciparum. Proc Natl Acad Sci U S A.

[B10] Scherf A, Hernandez-Rivas R, Buffet P, Bottius E, Benatar C, Pouvelle B, Gysin J, Lanzer M (1998). Antigenic variation in malaria: in situ switching, relaxed and mutually exclusive transcription of var genes during intra-erythrocytic development in Plasmodium falciparum. Embo J.

[B11] Freitas-Junior LH, Bottius E, Pirrit LA, Deitsch KW, Scheidig C, Guinet F, Nehrbass U, Wellems TE, Scherf A (2000). Frequent ectopic recombination of virulence factor genes in telomeric chromosome clusters of P. falciparum. Nature.

[B12] Hernandez-Rivas R, Hinterberg K, Scherf A (1996). Compartmentalization of genes coding for immunodominant antigens to fragile chromosome ends leads to dispersed subtelomeric gene families and rapid gene evolution in Plasmodium falciparum. Mol Biochem Parasitol.

[B13] Rasti N, Wahlgren M, Chen Q (2004). Molecular aspects of malaria pathogenesis. FEMS Immunol Med Microbiol.

[B14] Robinson BA, Welch TL, Smith JD (2003). Widespread functional specialization of Plasmodium falciparum erythrocyte membrane protein 1 family members to bind CD36 analysed across a parasite genome. Mol Microbiol.

[B15] Cheng Q, Cloonan N, Fischer K, Thompson J, Waine G, Lanzer M, Saul A (1998). stevor and rif are Plasmodium falciparum multicopy gene families which potentially encode variant antigens. Mol Biochem Parasitol.

[B16] Gardner MJ, Tettelin H, Carucci DJ, Cummings LM, Aravind L, Koonin EV, Shallom S, Mason T, Yu K, Fujii C, Pederson J, Shen K, Jing J, Aston C, Lai Z, Schwartz DC, Pertea M, Salzberg S, Zhou L, Sutton GG, Clayton R, White O, Smith HO, Fraser CM, Adams MD, Venter JC, Hoffman SL (1998). Chromosome 2 sequence of the human malaria parasite Plasmodium falciparum. Science.

[B17] Finn RD, Mistry J, Schuster-Bockler B, Griffiths-Jones S, Hollich V, Lassmann T, Moxon S, Marshall M, Khanna A, Durbin R, Eddy SR, Sonnhammer EL, Bateman A (2006). Pfam: clans, web tools and services. Nucleic Acids Res.

[B18] Florens L, Washburn MP, Raine JD, Anthony RM, Grainger M, Haynes JD, Moch JK, Muster N, Sacci JB, Tabb DL, Witney AA, Wolters D, Wu Y, Gardner MJ, Holder AA, Sinden RE, Yates JR, Carucci DJ (2002). A proteomic view of the Plasmodium falciparum life cycle. Nature.

[B19] Haeggstrom M, Kironde F, Berzins K, Chen Q, Wahlgren M, Fernandez V (2004). Common trafficking pathway for variant antigens destined for the surface of the Plasmodium falciparum-infected erythrocyte. Mol Biochem Parasitol.

[B20] Helmby H, Cavelier L, Pettersson U, Wahlgren M (1993). Rosetting Plasmodium falciparum-infected erythrocytes express unique strain-specific antigens on their surface. Infect Immun.

[B21] Petter M, Haeggstrom M, Khattab A, Fernandez V, Klinkert MQ, Wahlgren M (2007). Variant proteins of the Plasmodium falciparum RIFIN family show distinct subcellular localization and developmental expression patterns. Mol Biochem Parasitol.

[B22] Abdel-Latif MS, Dietz K, Issifou S, Kremsner PG, Klinkert MQ (2003). Antibodies to Plasmodium falciparum rifin proteins are associated with rapid parasite clearance and asymptomatic infections. Infect Immun.

[B23] Abdel-Latif MS, Khattab A, Lindenthal C, Kremsner PG, Klinkert MQ (2002). Recognition of variant Rifin antigens by human antibodies induced during natural Plasmodium falciparum infections. Infect Immun.

[B24] Marti M, Good RT, Rug M, Knuepfer E, Cowman AF (2004). Targeting malaria virulence and remodeling proteins to the host erythrocyte. Science.

[B25] Tham WH, Payne PD, Brown GV, Rogerson SJ (2007). Identification of basic transcriptional elements required for rif gene transcription. Int J Parasitol.

[B26] Abhiman S, Sonnhammer EL (2005). Large-scale prediction of function shift in protein families with a focus on enzymatic function. Proteins.

[B27] Knudsen B, Miyamoto MM (2001). A likelihood ratio test for evolutionary rate shifts and functional divergence among proteins. Proc Natl Acad Sci U S A.

[B28] Prim N, Bofill C, Pastor FI, Diaz P (2006). Esterase EstA6 from Pseudomonas sp. CR-611 is a novel member in the utmost conserved cluster of family VI bacterial lipolytic enzymes. Biochimie.

[B29] Stam MR, Danchin EG, Rancurel C, Coutinho PM, Henrissat B (2006). Dividing the large glycoside hydrolase family 13 into subfamilies: towards improved functional annotations of alpha-amylase-related proteins. Protein Eng Des Sel.

[B30] Khattab A, Klinkert MQ (2006). Maurer's clefts-restricted localization, orientation and export of a Plasmodium falciparum RIFIN. Traffic.

[B31] Kraemer SM, Smith JD (2003). Evidence for the importance of genetic structuring to the structural and functional specialization of the Plasmodium falciparum var gene family. Mol Microbiol.

[B32] DePristo MA, Zilversmit MM, Hartl DL (2006). On the abundance, amino acid composition, and evolutionary dynamics of low-complexity regions in proteins. Gene.

[B33] Enea V, Corredor V (1991). The evolution of plasmodial stage-specific rRNA genes is dominated by gene conversion. J Mol Evol.

[B34] Nielsen KM, Kasper J, Choi M, Bedford T, Kristiansen K, Wirth DF, Volkman SK, Lozovsky ER, Hartl DL (2003). Gene conversion as a source of nucleotide diversity in Plasmodium falciparum. Mol Biol Evol.

[B35] Posada D, Crandall KA (2002). The effect of recombination on the accuracy of phylogeny estimation. J Mol Evol.

[B36] Kennedy M, Holland BR, Gray RD, Spencer HG (2005). Untangling long branches: identifying conflicting phylogenetic signals using spectral analysis, neighbor-net, and consensus networks. Syst Biol.

[B37] Stiller JW, Hall BD (1999). Long-branch attraction and the rDNA model of early eukaryotic evolution. Mol Biol Evol.

[B38] Shakhnovich BE, Koonin EV (2006). Origins and impact of constraints in evolution of gene families. Genome Res.

[B39] Lavstsen T, Salanti A, Jensen AT, Arnot DE, Theander TG (2003). Sub-grouping of Plasmodium falciparum 3D7 var genes based on sequence analysis of coding and non-coding regions. Malar J.

[B40] Voss TS, Thompson JK, Waterkeyn J, Felger I, Weiss N, Cowman AF, Beck HP (2000). Genomic distribution and functional characterisation of two distinct and conserved Plasmodium falciparum var gene 5' flanking sequences. Mol Biochem Parasitol.

[B41] Fonager J, Cunningham D, Jarra W, Koernig S, Henneman AA, Langhorne J, Preiser P (2007). Transcription and alternative splicing in the yir multigene family of the malaria parasite Plasmodium y. yoelii: identification of motifs suggesting epigenetic and post-transcriptional control of RNA expression. Mol Biochem Parasitol.

[B42] Hiller NL, Bhattacharjee S, van Ooij C, Liolios K, Harrison T, Lopez-Estrano C, Haldar K (2004). A host-targeting signal in virulence proteins reveals a secretome in malarial infection. Science.

[B43] Golding GB, Dean AM (1998). The structural basis of molecular adaptation. Mol Biol Evol.

[B44] DePristo MA, Weinreich DM, Hartl DL (2005). Missense meanderings in sequence space: a biophysical view of protein evolution. Nat Rev Genet.

[B45] Weber JL (1988). Interspersed repetitive DNA from Plasmodium falciparum. Mol Biochem Parasitol.

[B46] Bozdech Z, Llinas M, Pulliam BL, Wong ED, Zhu J, DeRisi JL (2003). The transcriptome of the intraerythrocytic developmental cycle of Plasmodium falciparum. PLoS Biol.

[B47] Daily JP, Le Roch KG, Sarr O, Ndiaye D, Lukens A, Zhou Y, Ndir O, Mboup S, Sultan A, Winzeler EA, Wirth DF (2005). In vivo transcriptome of Plasmodium falciparum reveals overexpression of transcripts that encode surface proteins. J Infect Dis.

[B48] Le Roch KG, Zhou Y, Blair PL, Grainger M, Moch JK, Haynes JD, De La Vega P, Holder AA, Batalov S, Carucci DJ, Winzeler EA (2003). Discovery of gene function by expression profiling of the malaria parasite life cycle. Science.

[B49] Llinas M, Bozdech Z, Wong ED, Adai AT, DeRisi JL (2006). Comparative whole genome transcriptome analysis of three Plasmodium falciparum strains. Nucleic Acids Res.

[B50] Fernandez-Becerra C, Pein O, de Oliveira TR, Yamamoto MM, Cassola AC, Rocha C, Soares IS, de Braganca Pereira CA, del Portillo HA (2005). Variant proteins of Plasmodium vivax are not clonally expressed in natural infections. Mol Microbiol.

[B51] Preiser PR, Khan S, Costa FT, Jarra W, Belnoue E, Ogun S, Holder AA, Voza T, Landau I, Snounou G, Renia L (2002). Stage-specific transcription of distinct repertoires of a multigene family during Plasmodium life cycle. Science.

[B52] PlasmoDB v4.4. http://v4-4.plasmodb.org/.

[B53] Broad Institute of Harvard and M.I.T.. http://www.broad.mit.edu/.

[B54] Lassmann T, Sonnhammer EL (2005). Kalign--an accurate and fast multiple sequence alignment algorithm. BMC Bioinformatics.

[B55] Hall T (1999). BioEdit: a user-friendly biological sequence alignment editor and analysis program for Windows 95/98/NT. Nucl Acids Symp Ser.

[B56] GeneDB. http://www.genedb.org/.

[B57] Suyama M, Torrents D, Bork P (2006). PAL2NAL: robust conversion of protein sequence alignments into the corresponding codon alignments. Nucleic Acids Res.

[B58] Kumar S, Tamura K, Nei M (2004). MEGA3: Integrated software for Molecular Evolutionary Genetics Analysis and sequence alignment. Brief Bioinform.

[B59] Protein Sequence Logos and Relative Entropy. http://www.cbs.dtu.dk/~gorodkin/appl/plogo.html.

[B60] Schneider TD, Stephens RM (1990). Sequence logos: a new way to display consensus sequences. Nucleic Acids Res.

[B61] Bryson K, McGuffin LJ, Marsden RL, Ward JJ, Sodhi JS, Jones DT (2005). Protein structure prediction servers at University College London. Nucleic Acids Res.

[B62] Jones DT (1999). Protein secondary structure prediction based on position-specific scoring matrices. Journal of molecular biology.

[B63] Clamp M, Cuff J, Searle SM, Barton GJ (2004). The Jalview Java alignment editor. Bioinformatics (Oxford, England).

