# Supplementary figures and images for: Sub-grouping and sub-functionalization of the RIFIN multi-copy protein family
Source: BMC Genomics. 2008 Jan 15;9:19. doi: 10.1186/1471-2164-9-19 (PMC2257938; doi:10.1186/1471-2164-9-19)

## A-type

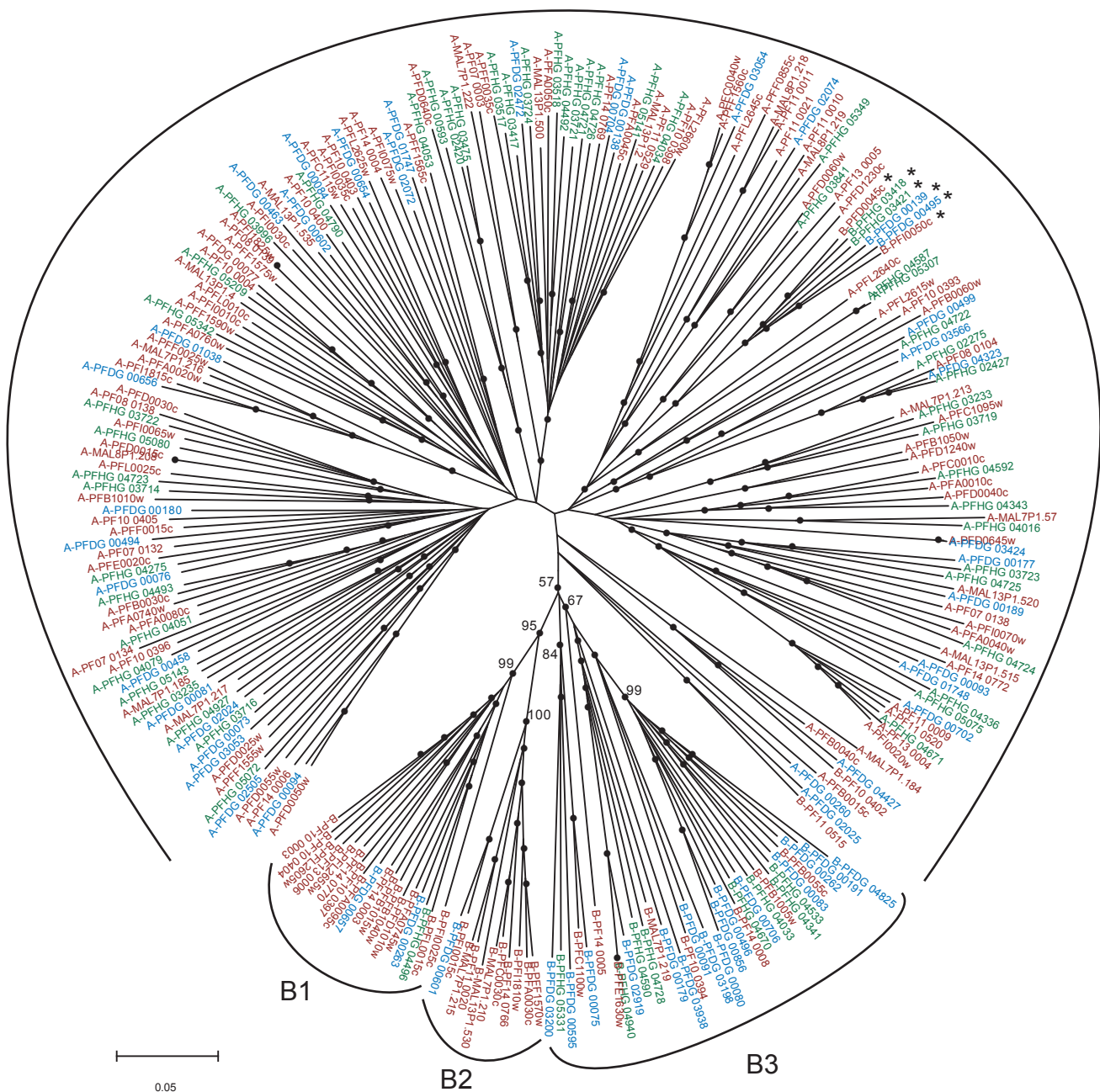

## B-types

Supplement: Additional file 1 — Phylogenetic tree of 3D7, DD2 and HB3 rif genes. The Neighbor Joining tree shows the segregation of A- and B-rif sequences (gaps considered as pairwise deletions). Stars show atypically grouped B-RIFIN sequences from all three strains. Colours: 3D7 sequences in red; DD2 sequences in blue; HB3 sequences in green. Bootstrap support, after 500 replicates, is only shown for major branches, dots at nodes indicate bootstrap values above or equal to 50%. [file 1471-2164-9-19-S1.pdf]

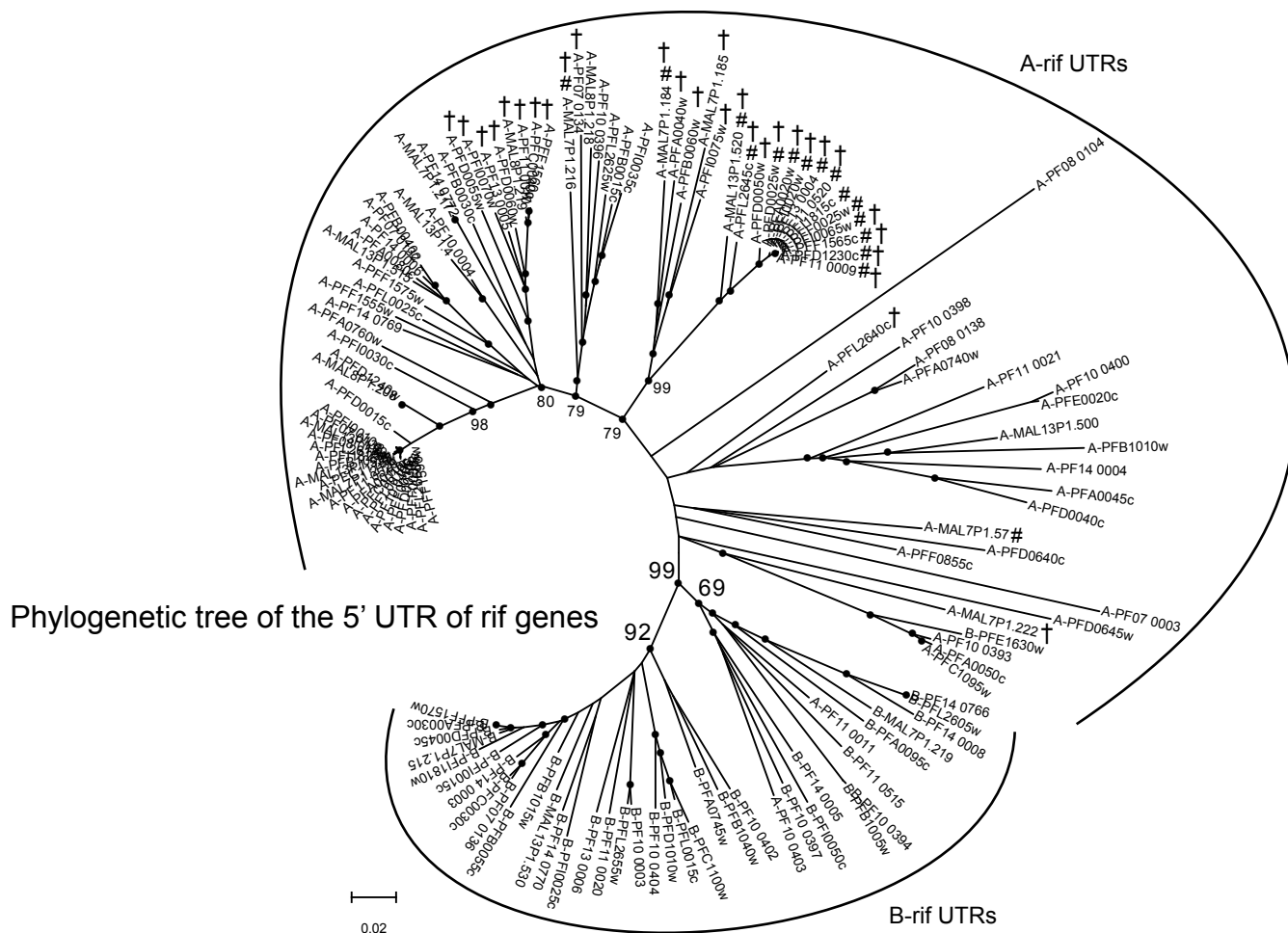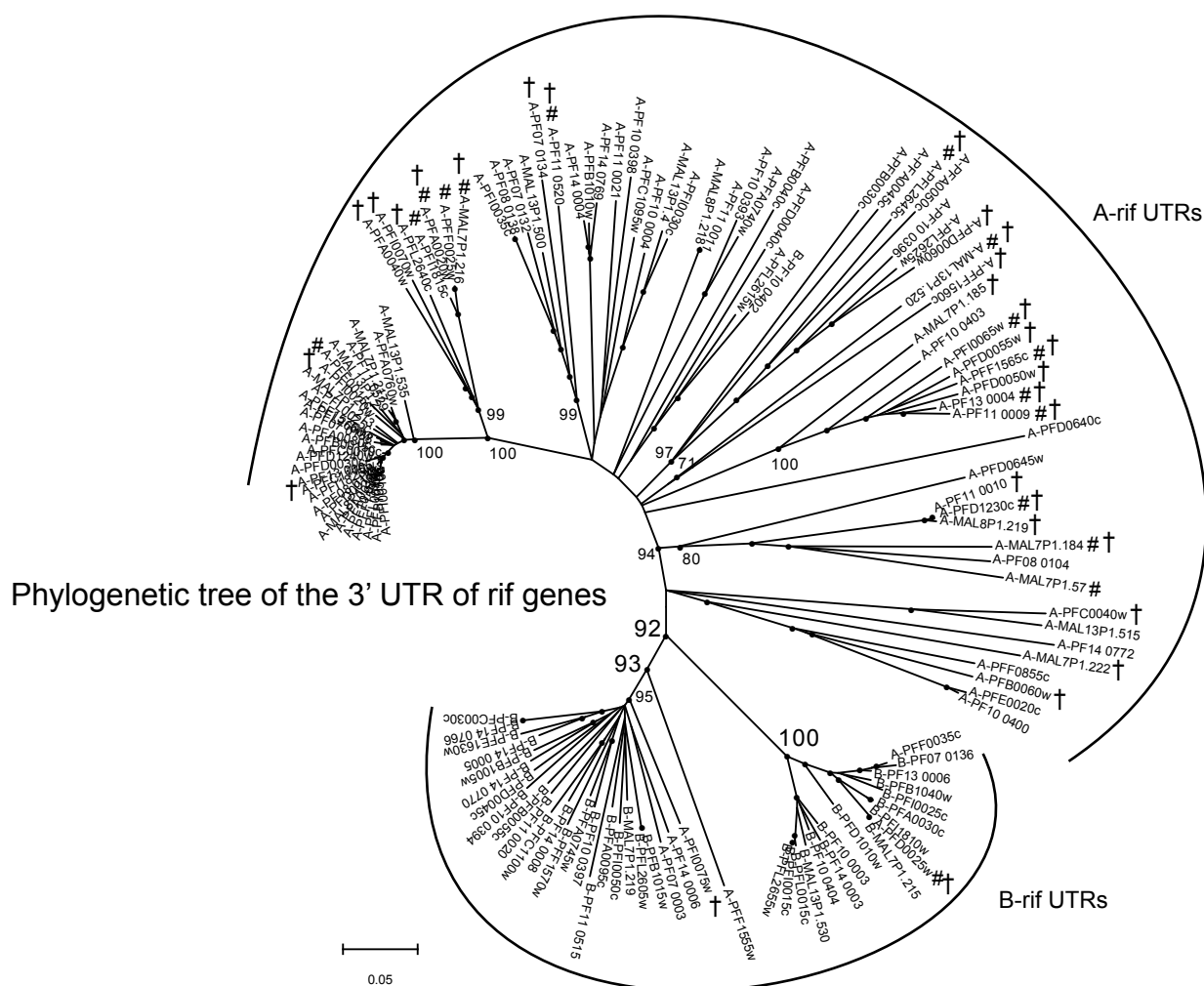

Supplement: Additional file 2 — Phylogenetic tree of 5' and 3' UTR sequences. The trees show the segregation of A- and B-rif UTRs (gaps considered as pairwise deletions). Bootstrap support, after 1000 replicates, is only shown for major branches, dots at nodes indicate bootstrap values above or equal to 60%. [file 1471-2164-9-19-S2.pdf]
